# Differential recruitment of coregulators to the *RORA* promoter adds another layer of complexity to gene (dys) regulation by sex hormones in autism

**DOI:** 10.1186/2040-2392-4-39

**Published:** 2013-10-11

**Authors:** Tewarit Sarachana, Valerie W Hu

**Affiliations:** 1 Department of Biochemistry and Molecular Medicine, The George Washington University School of Medicine and Health Sciences, 2300 I Street NW, Washington, DC 20037, USA; 2 Department of Clinical Chemistry, Faculty of Allied Health Sciences, Chulalongkorn University, 154 Rama I Road, Pathumwan, Bangkok 10330, Thailand

**Keywords:** Autism, RORA, Sex hormones, Nuclear receptor, Coregulator, Coactivator, Corepressor

## Abstract

**Background:**

Our independent cohort studies have consistently shown the reduction of the nuclear receptor RORA (retinoic acid-related orphan receptor-alpha) in lymphoblasts as well as in brain tissues from individuals with autism spectrum disorder (ASD). Moreover, we have found that RORA regulates the gene for aromatase, which converts androgen to estrogen, and that male and female hormones regulate *RORA* in opposite directions, with androgen suppressing RORA, suggesting that the sexually dimorphic regulation of *RORA* may contribute to the male bias in ASD. However, the molecular mechanisms through which androgen and estrogen differentially regulate *RORA* are still unknown.

**Methods:**

Here we use functional knockdown of hormone receptors and coregulators with small interfering RNA (siRNA) to investigate their involvement in sex hormone regulation of *RORA* in human neuronal cells. Luciferase assays using a vector containing various *RORA* promoter constructs were first performed to identify the promoter regions required for inverse regulation of *RORA* by male and female hormones. Sequential chromatin immunoprecipitation methods followed by quantitative reverse transcriptase-polymerase chain reaction (qRT-PCR) analyses of *RORA* expression in hormone-treated SH-SY5Y cells were then utilized to identify coregulators that associate with hormone receptors on the *RORA* promoter. siRNA-mediated knockdown of interacting coregulators was performed followed by qRT-PCR analyses to confirm the functional requirement of each coregulator in hormone-regulated *RORA* expression.

**Results:**

Our studies demonstrate the direct involvement of androgen receptor (AR) and estrogen receptor (ER) in the regulation of RORA by male and female hormones, respectively, and that the promoter region between −10055 bp and −2344 bp from the transcription start site of *RORA* is required for the inverse hormonal regulation. We further show that AR interacts with SUMO1, a reported suppressor of AR transcriptional activity, whereas ERα interacts with the coactivator NCOA5 on the *RORA* promoter. siRNA-mediated knockdown of *SUMO1* and *NCOA5* attenuate the sex hormone effects on *RORA* expression.

**Conclusions:**

AR and SUMO1 are involved in the suppression *RORA* expression by androgen, while ERα and NCOA5 collaborate in the up-regulation of *RORA* by estrogen. While this study offers a better understanding of molecular mechanisms involved in sex hormone regulation of *RORA*, it also reveals another layer of complexity with regard to gene regulation in ASD. Inasmuch as coregulators are capable of interacting with a multitude of transcription factors, aberrant expression of coregulator proteins, as we have seen previously in lymphoblasts from individuals with ASD, may contribute to the polygenic nature of gene dysregulation in ASD.

## Background

Autism spectrum disorder (ASD) is a neurodevelopmental disorder characterized by deficits in social understanding and interactions, aberrant communication, and repetitive, stereotyped behaviors, often with restricted interests [1,2]. Although the male-to-female ratios of ASD reported by different epidemiological studies are different depending on the populations studied [3-5], the prevalence of ASD is consistently higher in males than females, prompting theories that fetal or perinatal exposure to elevated levels of male hormones may increase susceptibility toward autism [6]. There is increasing evidence linking elevated fetal testosterone levels in amniotic fluid to autistic symptomatology [7,8] as well as morphology of the corpus callosum and sexually dimorphic brain regions [9,10]. Moreover, we have identified deregulated genes involved in androgen biosynthesis as well as higher testosterone levels in lymphoblastoid cell lines (LCL) from individuals with autism relative to their respective unaffected sex-matched siblings [11], further implicating a role for sex hormones in ASD, but there is still no clear understanding of the molecular mechanisms through which the sex hormones may play a role in autism susceptibility.

We have recently identified *RORA* as a novel candidate gene for ASD [12]. *RORA* encodes retinoic acid-related (RAR) orphan receptor-alpha, which is a ligand-dependent nuclear receptor that regulates gene transcription by binding to specific DNA response elements consisting of the consensus (A/G)GGTCA core motif in the regulatory region of target genes [13,14]. Our recent studies have demonstrated: reduced expression of *RORA* in LCL derived from individuals with autism [15]; increased methylation leading to reduced expression of *RORA* in the LCL from cases vs. sibling controls [12]; and decreased expression of RORA protein in the prefrontal cortex and the cerebellum of individuals with autism [12,16]. Together, these results link these molecular changes in RORA in blood-derived peripheral cells to molecular pathology in the brain tissues of individuals with ASD.

Studies using the Rora-deficient *staggerer* mouse model show that Rora is involved in several processes relevant to ASD, including Purkinje cell differentiation [17,18], cerebellar development [19,20], protection of neurons against oxidative stress [21], suppression of inflammation [22], and regulation of circadian rhythm [23]. Indeed, cerebellar abnormalities [24], including the loss of Purkinje cells [25], have been reported in autism, and the brain tissues of individuals with ASD show evidence of inflammation [26], as well as oxidative stress [27]. Moreover, there is increasing awareness of sleep disturbances in ASD [28-31], and genetic studies as well as our gene expression study of different subtypes of ASD have implicated a role for circadian rhythm regulator genes in ASD [15,32,33]. Behavioral studies on the *staggerer* mouse, primarily used as a model to study ataxia and dystonia [19], further show that these Rora-deficient mice also exhibit restricted behaviors reminiscent of autism, such as perseverative tendencies [34], limited maze patrolling [35], anomalous object exploration [36], as well as deficits in spatial learning [37]. Although there are currently no reported studies connecting social behaviors with Rora deficiency in mice, it is clear that RORA is associated with at least some of the symptomatology and pathology of ASD.

Recently, we found that RORA transcriptionally regulates multiple ASD-related genes, including *A2BP1*, *CYP19A1*, *HSD17B10, ITPR1*, *NLGN1*, and *NTRK2*, and reduced *RORA* expression leads to downregulation of these genes in human neuronal cells [38]. *CYP19A1* and *HSD17B10* respectively code for aromatase and hydroxysteroid (17β) dehydrogenase, which are enzymes responsible for the conversion of androgens to estradiol. Downregulation of either of these genes can lead to increased androgen and decreased estrogen, either of which may have a negative impact on brain development [39-41]. Our recent studies have further shown that *CYP19A1* and *HSD17B10* expression levels, as well as those of the other four confirmed gene targets of RORA, are significantly reduced in the frontal cortex of RORA-deficient autistic subjects relative to sex- and age-matched controls, and that aromatase protein levels are strongly correlated with RORA protein levels in the brain [16,38].

In addition to our finding that RORA regulates the transcription of genes involved in the enzymatic conversion of male to female hormones, neurohistological studies by other groups have reported that loss of Purkinje neurons in male Rora-deficient *staggerer* mice occurs much earlier in life in comparison to female *staggerer* mice [42,43], revealing a sexually dimorphic response to Rora deficiency. We recently demonstrated that male and female sex hormones inversely regulate *RORA* expression in human neuronal cells by suppressing and enhancing *RORA* expression, respectively [16]. These observations suggest that the pathology associated with RORA deficiency may be manifested in a sexually dimorphic manner which, in turn, may be related to the sex bias in ASD.

We have previously demonstrated hormone-mediated recruitment of androgen receptor (AR) and estrogen receptor alpha (ERα) to their respective binding sites on the *RORA* promoter [16]. Here we sought to determine whether AR and ERα are functionally involved in the regulation of *RORA.* Moreover, as it is known that hormone receptors must interact with other proteins (coregulators) to regulate transcription of their targets, we also sought to investigate the involvement of selected coregulator proteins in sex hormone-mediated regulation of RORA in human neuronal cells, with a focus on four coregulator proteins that were previously found to be differentially expressed in ASD [15].

## Methods

### Cell culture

The human neuroblastoma cells SH-SY5Y (ATCC, Manassas, VA, USA) were cultured in 1:1 MEM and Ham’s F12 media (Mediatech, Manassas, VA, USA) supplemented with 15% (v/v) fetal bovine serum (Atlanta Biologicals, Lawrenceville, GA, USA) and 1% penicillin/streptomycin (P/S; Mediatech). Cells were maintained at 37°C with 5% CO_2_, and split 1:2 every 3 to 4 days when the cells reached approximately 80% confluency. For harvesting, the cells were treated with trypsin-ethylenediaminetetraacetic acid (EDTA) (Mediatech) for 2 to 3 minutes to release them from the surface of the culture flask. Complete growth medium was then added to the flask containing suspension cells to inactivate trypsin. Cells were transferred to a sterile centrifuge tube and pelleted by spinning at 800 rpm for 5 minutes at 4°C and gently washed twice with ice-cold PBS. This study did not involve any human subjects or biomaterials taken from human subjects, and thus no ethical approval was required.

### Hormone treatment

For hormone treatment, SH-SY5Y cells were cultured in culture flasks until the cultures became approximately 80% confluent. Confluent cells were carefully washed twice with phenol red-free 1:1 DMEM/F12 media (Mediatech) supplemented with 15% charcoal dextran-treated serum (Atlanta Biologicals) and 1% P/S, and then cultured in the phenol red-free medium for 24 hours. Lyophilized 4,5α-dihydrotestosterone (DHT; Sigma-Aldrich, St. Louis, MO, USA) and 17β-estradiol (E2; Sigma-Aldrich) were diluted with molecular biology grade absolute ethanol (Fisher Scientific, Pittsburgh, PA, USA) to make 1 μM DHT or 1 μM E2 stock solutions. The stock solutions were further diluted with prewarmed complete phenol red-free culture medium to the desired final concentrations for hormone treatment and carefully added to the confluent cells. Cells were incubated in the hormone-supplemented phenol red-free medium at 37°C with 5% CO_2_ for 2 hours.

### siRNA transfection

siRNA-mediated knockdown of *AR*, *ERα*, *SUMO1*, or *NCOA5* was conducted using Lipofectamine RNAiMAX transfection agent (Invitrogen, Carlsbad, CA, USA) according to the manufacturer’s protocol. Briefly, SH-SY5Y cells were cultured in complete growth medium without antibiotics in a 6-well culture plate. When cells were approximately 50% confluent, the medium was substituted with phenol red-free culture medium without antibiotics and the cells were further incubated for 24 hours. siRNA (Santa Cruz Biotechnology, Dallas, TX, USA) targeting *AR*, *ERα*, *SUMO1*, or *NCOA5* (150 pmol) was diluted in 250 μl phenol red-free Opti-MEM I Reduced-Serum Medium (Invitrogen). Lipofectamine RNAiMAX (7.5 μl) was diluted in 250 μl phenol red-free Opti-MEM I reduced-serum medium in a separate tube. Then, the diluted siRNA and the diluted Lipofectamine RNAiMax were combined. The siRNA-Lipofectamine complex was incubated at room temperature for 5 minutes and added to the cells to a final siRNA concentration of 10 nM. The cells were incubated for 24 hours and then treated with 1 nM DHT, 1 nM E2, or ethanol (vehicle), for 2 hours according to the aforementioned hormone treatment procedure before harvesting for subsequent analysis. The list of siRNAs is shown in Additional file 1. Transfection efficiency was assessed by qRT-PCR analysis (Additional file 2).

### Quantitative RT-PCR analysis

Quantitative RT-PCR analyses were performed as described [11]. Total RNA from the cells was isolated using TRIzol (Invitrogen) and purified using the RNeasy Mini Kit (Qiagen, Valencia, CA, USA) following the manufacturers’ instructions. Human brain tissues were homogenized in the Bullet Blender Homogenizer (Next Advance, Averill Park, NY, USA) using nuclease-free glass beads, and total RNA was isolated from homogenized tissues using the RNeasy Mini Kit (Qiagen). RNA concentration was measured using a NanoDrop 1000 spectrophotometer (Thermo Scientific, Wilmington, DE, USA). A total of 1 μg of purified total RNA was used for cDNA synthesis using the iScriptcDNA Synthesis Kit (Bio-Rad, Hercules, CA, USA) according to the manufacturer’s protocols. The reaction was incubated at 25°C for 5 minutes, followed by 42°C for 30 minutes, and terminated at 85°C for 5 minutes. After reverse transcription, the cDNA reaction mixture was diluted to a volume of 50 μl with nuclease-free water and used as a template for qPCR analyses. Real-time PCR analyses were conducted using the Applied Biosystems 7300 Real-Time PCR System (Applied Biosystems, Foster City, CA, USA). Primers for qRT-PCR analyses (listed in Additional file 3) were designed using the Primer3 software [44] and synthesized by the Integrated DNA Technologies (Coralville, IA, USA). The relative quantity of transcripts in each sample was calculated using standard curves based on the relative quantity of 18S RNA transcript in 10-fold serial dilutions of that sample.

### Cloning

DNA was isolated from SH-SY5Y cells using the DNeasy Blood and Tissue Kit (Qiagen) according to the manufacturer’s protocols. The promoter regions of *RORA* were then amplified by a PCR method tailored for long stretches of nucleotides using the GoTaq Long PCR Master Mix (Promega, Madison, WI, USA) and DNA primers tagged with SfiI restriction sites at the 5′ end (Integrated DNA Technologies) according to the manufacturer’s protocols. Primers for PCR cloning are listed in Additional file 3. Briefly, a total of 0.5 μg of purified human genomic DNA isolated from SH-SY5Y cells was combined with the GoTaq Long PCR Master Mix and 10 μM (final concentration) of each DNA primer. The thermal cycling condition was set as follows: 95°C for 2 minutes, 30 cycles of 92°C for 30 seconds and 65°C for 15 minutes, followed by 72°C for 10 minutes. PCR products were analyzed by gel electrophoresis using 1% agarose. The bands with expected sizes were excised from the gel and purified using the Wizard SV Gel and PCR Clean-Up System (Promega).

Purified PCR products with different sizes were then separately inserted into pGEM-T Easy Vector (Promega) containing lacZ and ampicillin-resistant genes following the manufacturer’s instructions. The vector containing each PCR product was transformed into JM109 High-Efficiency Competent *E.coli* cells (Promega) by heat-shocking at exactly 42°C in a water bath for 50 seconds. Transformed bacteria were spread on duplicate Luria-Bertani LB agar plates containing 100 μg/ml ampicillin, 0.5 mM isopropyl-β-D-thio-galactoside (IPTG), and 80 μg/ml 5-bromo-4-chloro-indolyl-β-D-galactopyranoside (X-Gal), and incubated at 37°C overnight for blue-white screening. Well-isolated white colonies were selected and further cultured in LB medium supplemented with 125 μg/ml ampicillin at 37°C for 12 to 16 hours with shaking at 250 rpm.

Plasmid DNA was purified from bacteria using the Wizard Plus SV Minipreps DNA Purification System (Promega) according to the manufacturer’s protocol. Presence of *RORA* promoter in the plasmid was validated by long PCR analysis using GoTaq Long PCR Master Mix (Promega), followed by gel electrophoresis. *RORA* promoter inserts were then released from the pGEM-T Easy plasmids using SfiI restriction enzyme and purified by gel electrophoresis. The luciferase vector pGL4.20[luc2/Puro] (Promega) containing firefly luciferase, puromycin-resistant, and ampicillin-resistant genes was prepared by digestion with the SfiI restriction enzyme and dephosphorylation using TSAP Thermosensitive Alkaline Phosphatase (Promega) to prevent self-recircularization of the linearized vector during ligation. *RORA* promoter inserts were then ligated into the dephosphorylated luciferase vector using LigaFast Rapid DNA Ligation System (Promega) and transformed into the JM109 High-Efficiency Competent *E.coli* cells. Transformed bacteria were cultured on LB agar plate containing 125 μg/ml ampicillin. Colonies of bacteria were harvested and further cultured in LB medium containing 125 μg/ml ampicillin overnight. Luciferase plasmids containing the *RORA* promoter regions were then purified from the transformed bacteria using Wizard Plus SV Minipreps DNA Purification System (Promega). Presence of *RORA* promoter insert was confirmed by long PCR analysis.

### Dual-luciferase reporter assays

The pGL4.20[luc2/Puro] vector containing a specific *RORA* promoter region was mixed with the pGL4.74[hRluc/TK] vector containing *Renilla reniformis* luciferase gene with a ratio of 50:1 in phenol red-free Opti-MEM I reduced-serum medium (Invitrogen). The FuGENE HD Transfection Reagent (Promega) was then added to the medium containing the vectors to obtain a ratio of 3:1 (that is, 3 μl transfection agent for 1 μg DNA). The mixture was added to a 96-well plate containing SH-SY5Y cells approximately 2 × 10^4^ cells/well) and incubated at 37°C, 5% CO_2_, for 48 hours. The transfected cells were treated with 10 nM DHT, 10 nM E2, or ethanol control for 2 hours, then dual-luciferase reporter assays (duplicates) were performed using the Dual-Luciferase Reporter Assay System (Promega) according to the manufacturer’s protocol. Briefly, lysis buffer was added to the 96-well plates containing hormone-treated transfected cells and complete lysis of cells was assessed under an inverted microscope. Cell lysates were collected and transferred to Cellstar 96-well plate (BioExpress, Kaysville, UT, USA). A Veritas Microplate Luminometer (Turner Biosystems, Sunnyvale, CA, USA) was used for detection of firefly and Renilla luminescence as well as for measurement of both firefly and Renilla luciferase activity signals. Firefly luciferase luminescence in each well was normalized by Renilla luciferase luminescence in the same well.

### Prediction of transcription factor binding elements

Putative binding sites of AR and ERα in the human *RORA1* promoter region and putative binding sites of RORA in the promoter regions of *CYP19A1* were predicted using PROMO 3.0 [45,46], JASPAR [47], and SABiosciences EpiTect ChIP Search Portal (SABiosciences, Valencia, CA, USA) programs. For each gene, a total of three to four predicted transcription factor binding sites (listed in Additional file 4) were selected for chromatin immunoprecipitation analyses.

### Chromatin immunoprecipitation

Chromatin was isolated from SH-SY5Y cells and sheared using the ChIP-IT Express Enzymatic Kit (Active Motif, Carlsbad, CA, USA) according to the manufacturer’s instructions. Briefly, confluent SH-SY5Y cells (approximately 1.5 × 10^7^ cells in a T-175 flask) were fixed with 10% formaldehyde for exactly 5 minutes and the fixation reaction was stopped by adding 10% glycine. The cells were washed with 10 ml ice-cold PBS for 5 seconds, then 6 ml ice-cold PBS supplemented with 0.5 mM (final concentration) phenylmethylsulfonyl fluoride (PMSF) supplied in the kit was added to the culture flask to wash and chill the cells. The crosslinked cells were transferred from the flask to a pre-chilled centrifuge tube by scraping gently with a cell scraper. Crosslinked cells were homogenized by douncing 40 to 50 times on ice using a dounce homogenizer with a tight pestle to release the nucleus. Optimal cell lysis was assessed under a phase contrast microscope using a hemacytometer. The cell lysate was transferred to a 1.7 ml microcentrifuge tube and centrifuged for 10 minutes at 5,000 rpm (2,400 RCF) in a 4°C microcentrifuge to pellet nuclei. Chromatin was then isolated from the nuclear pellets and sheared into 150 to 1,000 bp fragments by incubating with 10 U/ml (final concentration) Enzymatic Shearing Cocktail (Active Motif) at 37°C for exactly 10 minutes. The enzymatic shearing reaction was stopped by adding EDTA to a final concentration of 10 mM EDTA and chilling the reaction tube on ice for 10 minutes. To assess shearing efficiency and determine DNA concentration in the sheared chromatin, a 50 μl aliquot of each sheared chromatin sample was reverse-crosslinked by mixing with 150 μl nuclease-free water and 10 μl 5 M NaCl. The reaction was incubated at 65°C in a water bath overnight. After incubation, 1 μl RNaseA (10 μg/μl) was added to each tube and the reaction was incubated at 37°C for 15 minutes. The reaction was then mixed with 10 μl Proteinase K (0.5 μg/μl) and further incubated at 42°C for 1.5 hours. The reverse-crosslinked DNA was isolated using standard phenol/chloroform extraction technique and purified using the Chromatin IP DNA Purification Kit (Active Motif). DNA concentration was measured using a NanoDrop 1000 spectrophotometer (Thermo Scientific, Wilmington, DE, USA). Optimal shearing was assessed by agarose gel electrophoresis. For chromatin immunoprecipitation reaction, the remaining enzymatically sheared, non-reverse-crosslinked chromatin was aliquoted into multiple tubes, each of which contained approximately 25 μg chromatin DNA.

Each aliquot of chromatin was then used as input chromatin for sequential immunoprecipitation according to the manufacturer’s protocol for the Re-ChIP-IT Kit (Actif Motif). For each reaction, sheared chromatin (approximately 25 μg per reaction) was first immunoprecipitated by mixing with 1 μg of anti-AR, anti-ERα, anti-RORA, or IgG antibody and 25 μl Protein G Magnetic Beads (Active Motif). The reaction was then incubated on an end-to-end rotator overnight at 4°C. After incubation, the immunoprecipitated chromatin was eluted from the magnetic beads using the Re-ChIP-IT Elution Buffer (Active Motif) and desalted using the Active Motif Desalting Columns to remove the first antibody on the chromatin. Then, 1 μg of the second antibody (anti-NCOA1, anti-NCOA5, anti-SUMO1, anti-FHL2, or IgG antibody) and 25 μl of the LSV Protein G Magnetic Beads (Active Motif) were added to the desalted chromatin (approximately 90 μl) and incubated on an end-to-end rotator overnight at 4°C to re-immunoprecipitate the chromatin. To validate that successful re-immunoprecipitation was caused by the second antibody and not by carried over first antibody, a re-immunoprecipitation reaction without the second antibody (that is, no-second-antibody control) was also performed in parallel and included in subsequent qPCR analysis. After incubation, DNA from re-immunoprecipitated chromatin was isolated and purified using the ChIP DNA Purification Kit (Active Motif). The list of antibodies for sequential ChIP is shown in Additional file 1.

### Real-time, quantitative PCR analysis of immunoprecipitated DNA

Real-Time qPCR analysis was conducted using Applied Biosystems 7300 Real-Time PCR System (Applied Biosystems) to determine the enrichment of each AR/ERα/RORA binding element in immunoprecipitated or sequentially immunoprecipitated DNA. Primers for qPCR analysis were designed using Primer3 software [44] and synthesized by Integrated DNA Technologies (IDT). Input DNA was diluted into five 10-fold serial dilutions and included in the qPCR analysis. Relative enrichment values of AR/ERα/RORA binding elements in each sequentially immunoprecipitated chromatin were calculated using standard curves obtained from the enrichment of respective AR/ERα/RORA binding elements in the 10-fold serial dilutions of input DNA. The list of primers is shown in Additional file 3.

### Co-immunoprecipitation analysis

The SH-SY5Y cells were cultured in complete growth medium until the confluency was approximately 70 to 80% and hormone treatment was conducted as mentioned above. Co-immunoprecipitation (co-IP) assays were then conducted using Pierce Crosslink Magnetic IP/Co-IP Kit (Thermo Scientific) according to the manufacturer’s protocol, using antibodies against four coregulator proteins that were found to be differentially expressed in LCL from individuals with ASD relative to that of unaffected controls ([15]; see Additional file 5). Briefly, the medium was removed from the flask containing cells. Then, cells were washed with ice-cold PBS containing phosphatase and deacetylase inhibitors and whole-cell lysis buffer was added directly into the flask. Protein A/G magnetic beads for immunoprecipitation were treated with anti-NCOA1, anti-NCOA5, anti-SUMO1, anti-FHL2, or nonspecific IgG antibody, and the antibody-bound magnetic beads were crosslinked with 20 μM disuccinimidyl suberate. The list of antibodies used is shown in Additional file 1. The crosslinked magnetic beads were mixed with SH-SY5Y whole-cell extract and incubated overnight at 4°C. The magnetic beads were then collected and protein complexes bound to the beads were eluted. Eluted immunoprecipitated proteins were used for subsequent western blot analysis as described below to determine the enrichment of AR, ERα, or RORA protein.

### BCA assays

Protein concentration was determined by BCA assays using Pierce BCA Protein Assay Kit (Thermo Scientific) according to the manufacturer’s directions for microplate assays. Briefly, the sample was mixed with BCA reagent containing bicinchoninic acid (BCA) and cupric sulfate and incubated at 37°C for 30 minutes. To determine protein concentration in an unknown sample, serial dilutions of bovine serum albumin (25 to 2,000 μg/ml) were included in the analysis and used for creating a standard curve. The absorbance of each sample was measured at 562 nm using a Synergy HT Multi-Mode microplate reader (BioTek, Winooski, VT, USA). The protein concentration in each unknown sample was calculated using standard curves obtained from absorbance values of the serial dilutions of albumin standards.

### Western blot analysis

A total of 30 μg of protein was mixed with 5× Thermo Scientific Lane Marker Non-Reducing Sample Buffer (Thermo Scientific) containing 0.3 M Tris–HCl, 5% SDS, 50% glycerol, and pink tracking dye. The sample was boiled for 5 minutes and loaded onto a Mini-PROTEAN TGX precast polyacrylamide gel (Bio-Rad, Hercules, CA, USA). Electrophoresis was conducted at 200 V using 1× Tris-glycine buffer containing 25 mM Tris base, 190 mM glycine, and 0.1% SDS, as a running buffer. Proteins on the gel were then transferred to polyvinylidene fluoride (PVDF) membrane and blocking was performed for 1 hour at 4°C using 5% (w/v) non-fat dry milk (Bio-Rad) in Tris-buffered saline and Tween 20 (TBST) buffer containing 2.42 g Trizma-HCl, 8 g NaCl, and 1× Tween 20. Protein detection was conducted by incubating the PVDF membrane with anti-AR, anti-ERα, anti-RORA, or anti-RORA1 antibody (1:200 in 1% milk/TBST) at 4°C overnight. The membrane was washed and treated with donkey secondary antibody conjugated with horseradish peroxidase (HRP; Santa Cruz Biotechnology; 1:2,500) for 1 hour at room temperature. Protein visualization was performed using a chemiluminescence method by incubating the membrane in chemiluminescence substrates (PerkinElmer, Waltham, MA, USA). Protein signals on the membrane were detected using a ChemiDoc XRS+ Imager (Bio-Rad).

### Statistical analyses

The two-tailed Student *t* test was used to determine the statistical significance of differences in the means of two groups. A *P* value of less than 0.05 was considered statistically significant. For comparisons of the means of three or more groups, ANOVA followed by post hoc *t* tests were conducted using the StatPac (Pepin, WI, USA) statistics calculator. A *P* value of less than 0.05 was considered statistically significant.

## Results

### AR and ERα are required for sex hormone regulation of *RORA*

**Figure 1:**
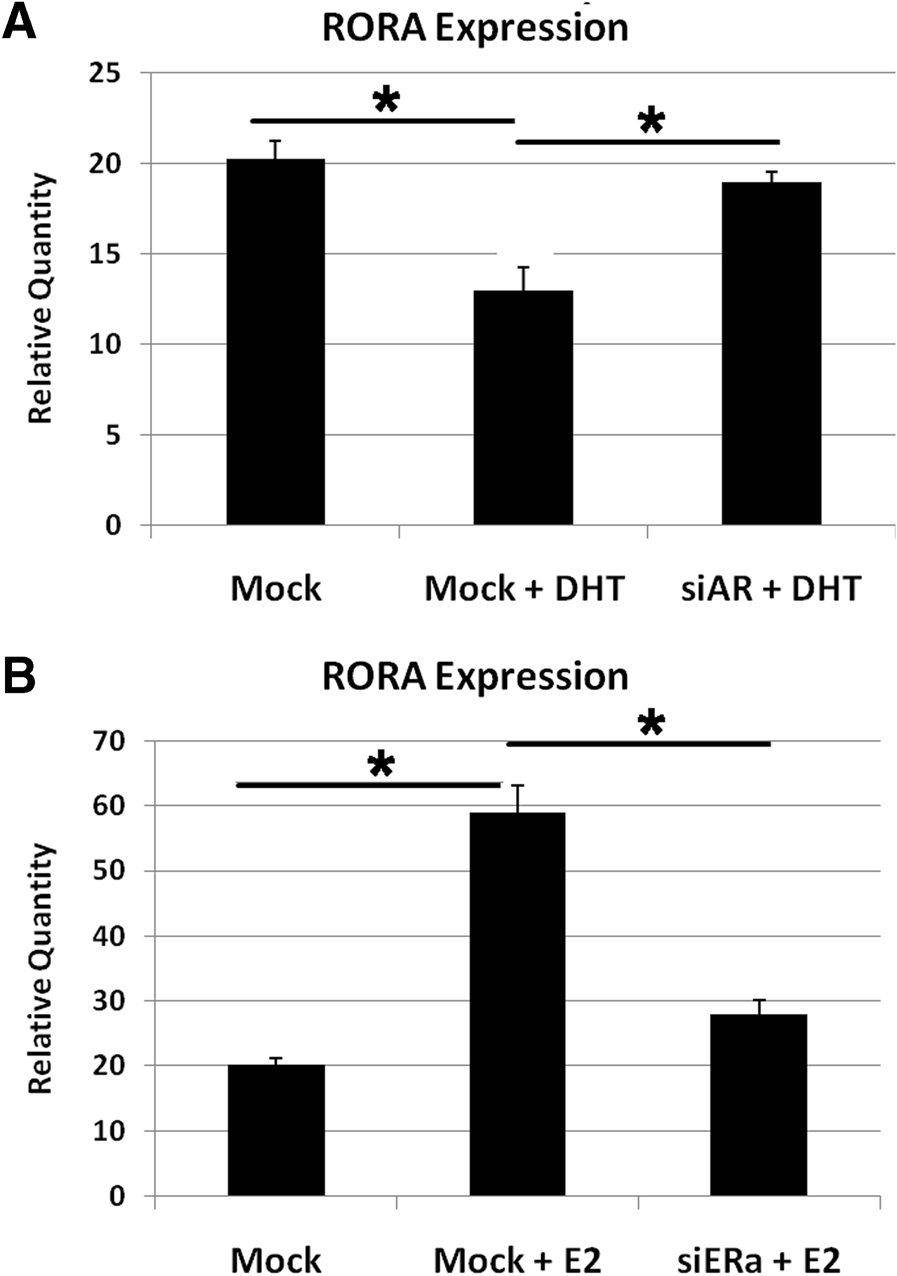
**siRNA-mediated knockdown of AR and ERα reveals direct role of hormone receptors in regulation of *RORA *expression by sex hormones.** SH-SY5Y cells were transfected with siAR **(A)**, siERα **(B)**, or vehicle control (mock-transfected) for 24 hours, then treated with 1 nM DHT **(A)**, 1 nM E2 **(B)**, or ethanol for 2 hours. Quantitative RT-PCR analysis (n = 3) of the hormone-treated, transfected cells was conducted to determine *RORA* expression. Relative *RORA* quantity in each sample was calculated using a standard curve obtained from *18S* expression levels in 10-fold serial dilutions of that sample. Error bars indicate SEM. Statistical significance of the differences between groups was determined by ANOVA (*P* <0.01 for each knockdown experiment) followed by post hoc *t* tests. *P <0.05. AR, androgen receptor; DHT, 4,5α-dihydrotestosterone; E2, 17β-estradiol; ERα, estrogen receptor alpha; SEM, standard error of the mean.

We have recently demonstrated that AR and ERα are recruited to the *RORA* promoter region in the presence of DHT and E2, respectively [16]. However, androgens and estrogens are also capable of regulating their transcriptional targets through AR- and ERα-independent mechanisms. To determine whether DHT mediates its repressive effect on *RORA* expression through AR, we transfected the human neuronal cells SH-SY5Y with siRNA against AR (siAR) prior to treatment with DHT for 2 hours. *RORA* expression, measured by qRT-PCR analysis, was compared with that in mock-transfected cells treated with DHT or ethanol. Unlike the DHT-treated mock control cells, which exhibited a significant decrease in *RORA* expression, the expression of *RORA* in the siAR-transfected cells treated with DHT was not significantly changed in comparison with mock-transfected cells treated with ethanol (Figure 1A), indicating that AR is required for DHT-mediated repression of *RORA*.

To determine whether ERα is required for E2-mediated upregulation of *RORA*, we transfected the cells with siERα before treatment with E2 following the same protocol used for the aforementioned AR knockdown. Figure 1B shows that the increase in *RORA* expression in response to E2 was significantly attenuated (though not completely abolished) in the siERα-transfected cells in comparison with the mock-transfected cells, indicating that ERα is involved in the upregulation of *RORA* mediated by E2. These results led us to further investigate the molecular mechanisms involved in sex hormone regulation of RORA through AR and ERα.

### Androgen and estrogen require distal AR/ERα promoter binding elements to inversely modulate *RORA*

**Figure 2:**
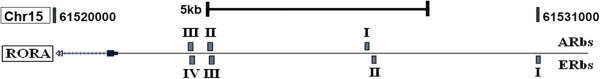
**Schematic diagram showing the upstream region of the *RORA *gene (edited from the UCSC Genome Browser).** Potential AR and ER binding sites are labeled (ARbs, = AR potential binding site; ERbs, = ER potential binding site). AR, androgen receptor; ER, estrogen receptor.

AR and ERα are able to regulate transcription by binding directly to specific DNA elements in the promoter region of their target genes as well as by non-genomic mechanisms. The *RORA* promoter region contains multiple binding sites for AR and ER spanning a region as far as approximately 10 kb upstream of the transcription start site (TSS). In our previous study, we selected four ER binding sites (ERbs-I, -II, -III, and IV) and three AR binding sites (ARbs-I, -II, and -III) located within 10 kb upstream of the TSS of RORA for chromatin immunoprecipitation analysis of hormone receptor binding (Figure 2). We found that AR and ERα are recruited to some of these binding elements in the presence of DHT and E2, respectively [16]. However, it is still unclear which binding sites are involved in the DHT-mediated downregulation and E2-mediated upregulation of *RORA*. We therefore constructed several firefly luciferase vectors containing different AR/ERα binding sites in the region upstream of the TSS and then conducted dual luciferase reporter assays of promoter activity in SH-SY5Y cells treated with DHT, E2, or ethanol, using the co-transfected Renilla luciferase vector as a negative control. The firefly luminescence signal in each reaction was normalized with the signal from Renilla luciferase in the same reaction to account for the variability between transfection experiments.

Interestingly, the firefly luciferase gene was oppositely regulated by DHT and E2 in the directions previously observed with endogenous *RORA* only when the gene was driven by the longest *RORA* promoter region (−10055 to −48) containing ARbs-I-III and ERbs-I-IV (Table 1). Consistent with the findings from our previous study [16], DHT significantly suppressed *RORA* promoter-driven luciferase activity (*P* value <0.05), whereas E2 enhanced it by over 2.5 fold (*P* value <0.05). When ERbs-I was deleted, the enhancing effect of E2 on *RORA* promoter activity was completely diminished and, instead, the luciferase activity was significantly suppressed by E2. This finding indicates that the ERbs-I is critical for the upregulation of *RORA* transcription by E2. Without ERbs-I, E2 has a negative effect on *RORA* promoter activity.

**Table 1:** **Fold-change in *RORA *** **promoter-driven luciferase activity in response to DHT or E2**

**Region of RORA promoter fused to luciferase (bp from TSS)**	**AR and ER binding sites in promoter region**	Fold-change^*^with DHT (*P * **value**^**¥**^**)**	Fold-change^*^with E2 (*P * **value**^**¥**^**)**
−48 to −10055	ARbs-I-III; ERbs-I-IV	0.78 (0.020)	2.29 (0.021)
−48 to −6000	ARbs-I-III; ERbs-II-IV	0.81 (0.008)	0.71 (0.001)
−48 to −2344	ARbs-II-III; ERbs-II-IV	1.22 (0.024)	0.86 (0.035)
−48 to −1992	ARbs-III; ERbs-IV	0.83 (0.007)	0.74 (0.001)

*Fold-change relative to vehicle treatment; ^¥^two-tailed *t* test. DHT, 4,5α-dihydrotestosterone; E2, 17β-estradiol; AR, androgen receptor; ERα, estrogen receptor alpha; ARbs, AR potential binding site; ERbs, ER potential binding site.

SH-SY5Y cells were transfected with expression vectors containing different *RORA* promoter constructs fused to the firefly luciferase gene together with a second expression vector containing the Renilla luciferase gene, at a ratio of 50:1. After a 2-hour treatment with the indicated hormone or vehicle (ethanol), firefly luciferase luminescence was determined for each sample and normalized by Renilla luminescence in the same sample. *T* tests were performed to determine the significance of the differences between hormone-treated and ethanol-treated samples. There were no significant differences in firefly luciferase activity between hormone-treated and vehicle-treated samples when the cells were transfected with the empty vector (that is, no RORA promoter).

In the presence of all AR binding sites in the *RORA* promoter region, the luciferase activity was significantly suppressed by DHT (Table 1). When ARbs-I was deleted, DHT significantly enhanced, rather than suppressed, promoter activity (*P* value <0.01). However, when both ARbs-I and -II were deleted, the suppressive effect of DHT on *RORA* promoter-driven luciferase activity was restored. This finding indicates that DHT can induce suppression of *RORA* promoter activity through ARbs-I and ARbs-III, but enhancement of the promoter activity through ARbs-II. These data indicate that the promoter region between −2344 and −10055 upstream of the *RORA* TSS which contains both ARbs-I and ERbs-I is required for DHT-mediated downregulation and E2-mediated upregulation of *RORA*.

### Identification of AR and ERα coregulators involved in sex hormone regulation of *RORA*

Hormone receptors such as AR and ERα must associate with coregulator proteins to regulate expression of their transcriptional targets. Although a number of AR and ERα coregulator proteins have been identified elsewhere, it is unknown which coregulators are involved in sex hormone regulation of *RORA*, particularly in the context of neuronal cells. We thus sought to identify coregulator proteins that interact specifically with AR and ERα at ARbs-I and ERbs-I, respectively.

**Figure 3:**
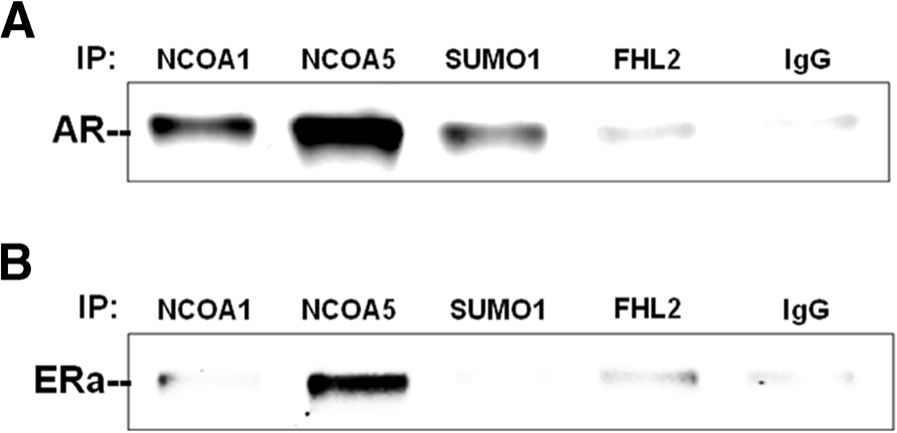
**Co-immunoprecipitation analysis of AR/ERα coregulators.** Whole-cell lysates of SH-SY5Y cells treated with 1nM DHT **(A)** or 1nM E2 **(B)** for 2 hours were immunoprecipitated with anti-NCOA1, anti-NCOA5, anti-SUMO1, anti-FHL2, or IgG antibody. Western blot analyses were performed to determine the enrichment of AR **(A)** and ERα **(B)** in the immunoprecipitated complexes. AR, androgen receptor; DHT, 4,5α-dihydrotestosterone; E2, 17β-estradiol; ERα, estrogen receptor alpha.

As mentioned earlier, we found a number of nuclear receptor coregulators differentially expressed in LCL derived from individuals with ASD relative to sex-matched typically developing individuals [15]. These coregulators included NCOA1, NCOA5, SUMO1, and FHL2, with known associations with AR and ERα. To determine whether these coregulators interact with AR in human neuronal cells, co-immunoprecipitation analyses were performed using whole-cell lysates of DHT-treated SH-SY5Y cells and anti-NCOA1, anti-NCOA5, anti-SUMO1, anti-FHL2, or nonspecific IgG antibody. Western blot analysis showed that AR was clearly enriched in protein samples immunoprecipitated with antibodies to NCOA1, NCOA5, and SUMO1, with only marginal enrichment with antibody to FHL2, in comparison with AR in the IgG-immunoprecipitated sample (Figure 3A), indicating that AR is capable of interacting with these coregulators in the human neuronal cell line. Similar co-immunoprecipitation analyses using cells treated with E2 showed an increase in the enrichment of ERα protein in protein samples co-immunoprecipitated with NCOA5 and, to a lesser extent, with FHL2 (Figure 3B), indicating that ERα interacts with these two coregulators in the human neuronal cell line SH-SY5Y.

**Figure 4:**
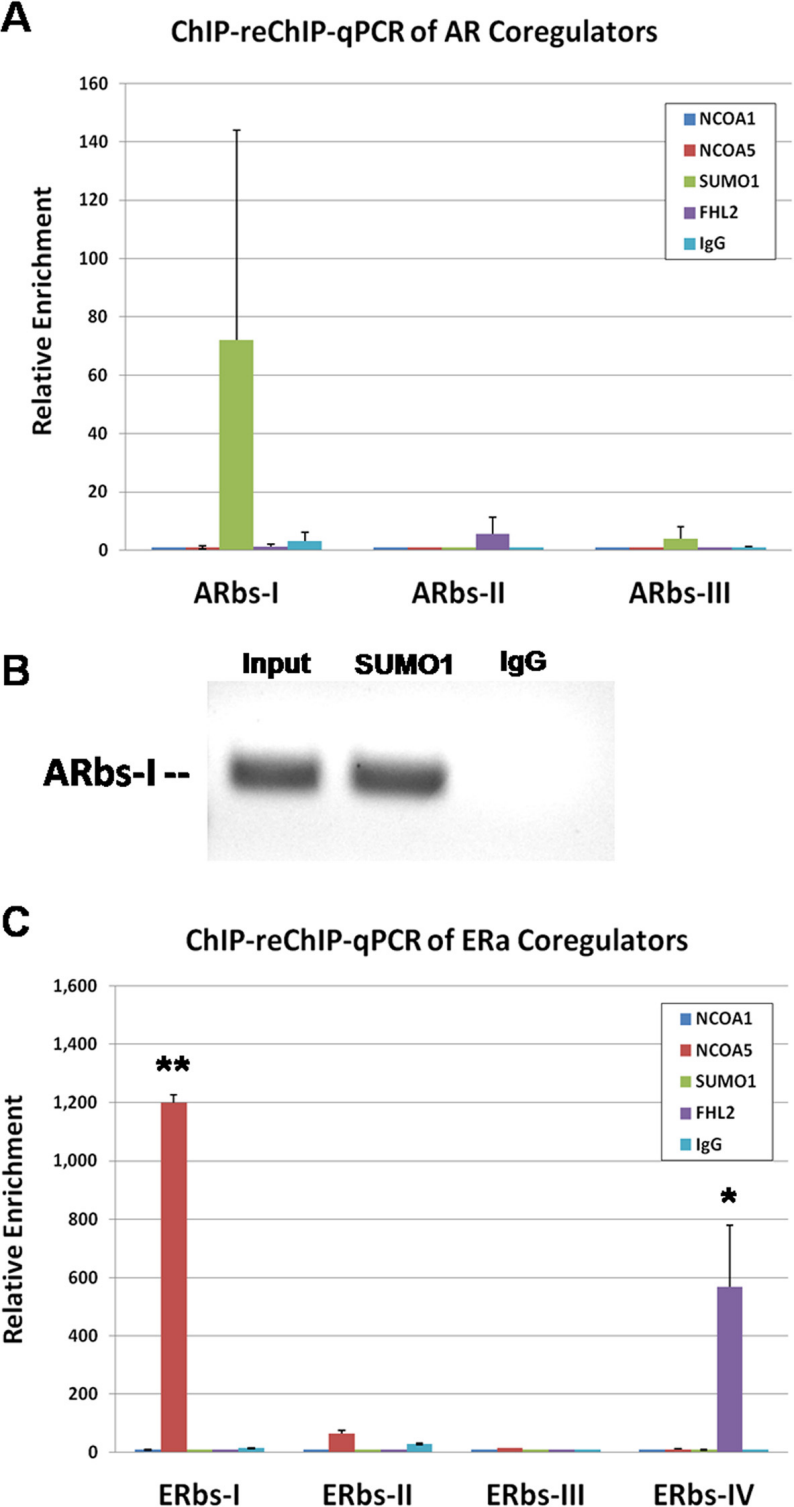
**ChIP-reChIP analyses identify coregulators that associate with AR/ERα on the *RORA *promoter. (A)** SH-SY5Y cells were treated with 1 nM DHT and whole-cell lysates were prepared and immunoprecipitated with anti-AR or IgG antibody. The immunoprecipitated chromatin-antibody complex was then dissociated and washed to remove the antibody. The immunoprecipitated chromatin was then re-immunoprecipitated using anti-NCOA1, anti-NCOA5, anti-SUMO1, anti-FHL2, nonspecific IgG antibody, or no-second-antibody negative control. The enrichment of AR binding sites in the *RORA* promoter region in each re-ChIP sample was then determined using qPCR analysis (n = 3) and normalized with reference to the no-second-antibody control. **(B)** Gel electrophoresis analysis of PCR products using input chromatin, chromatin immunoprecipitated with anti-AR followed by anti-SUMO1 antibody, or IgG followed by IgG, as templates. PCR primers were designed to specifically amplify ARbs-I. **(C)** ChIP-reChIP analysis of coregulators associated with ERα binding sites in *RORA* promoter was conducted in the same manner as for AR binding sites using SH-SY5Y cells treated with 1nM E2. Error bar indicates SEM. **P <0.01, *P <0.05. AR, androgen receptor; ChIP-reChIP, sequential chromatin immunoprecipitation; DHT, 4,5α-dihydrotestosterone; E2, 17β-estradiol; ERα, estrogen receptor alpha; SEM, standard error of the mean.

To further determine whether these coregulators are involved in AR-mediated regulation of *RORA* in human neuronal cells, sequential chromatin immunoprecipitation (ChIP-reChIP) analysis of SH-SY5Y cell lysates was conducted using anti-AR antibody, followed by each of the anti-coregulator antibodies in separate reactions. The enrichment of AR binding sites in the *RORA* promoter region was then determined by qPCR analysis of the reChIP samples. An increase in the average enrichment of ARbs-I was observed in the chromatin sample sequentially immunoprecipitated with antibody to AR, followed by antibody to SUMO1 (Figure 4A). Because there was a high degree of variability in the enrichment of ARbs-I in SUMO1 re-immunoprecipitated chromatin, which is probably due to low expression of AR in the SH-SY5Y cells, we conducted PCR using DNA resulting from the sequential immunoprecipitation and primers designed to amplify ARbs-I, and then visualized the PCR product by gel electrophoresis. As shown in Figure 4B, ARbs-I was enriched in the product that resulted from the sequential ChIP with AR and SUMO1 antibodies, in comparison with that resulting from pull down with nonspecific IgG. This finding confirms that AR interacts with SUMO1 at the AR binding element ARbs-I in the *RORA* promoter region.

ChIP-reChIP analysis of coregulators associated with ERα at its receptor binding sites in the *RORA* promoter was conducted in the same manner as for AR binding sites using SH-SY5Y cells treated with E2. Figure 4C shows a significant increase in the enrichment of ERbs-I in the reChIP reaction with anti-NCOA5, while ERbs-IV was significantly enriched when antibody against FHL2 was used for the second ChIP. This finding indicates that ERα interacts with NCOA5 at ERbs-I and FHL2 at ERbs-IV on the *RORA* promoter.

### Regulation of *RORA* by sex hormones is mediated by SUMO1 and NCOA5

**Figure 5:**
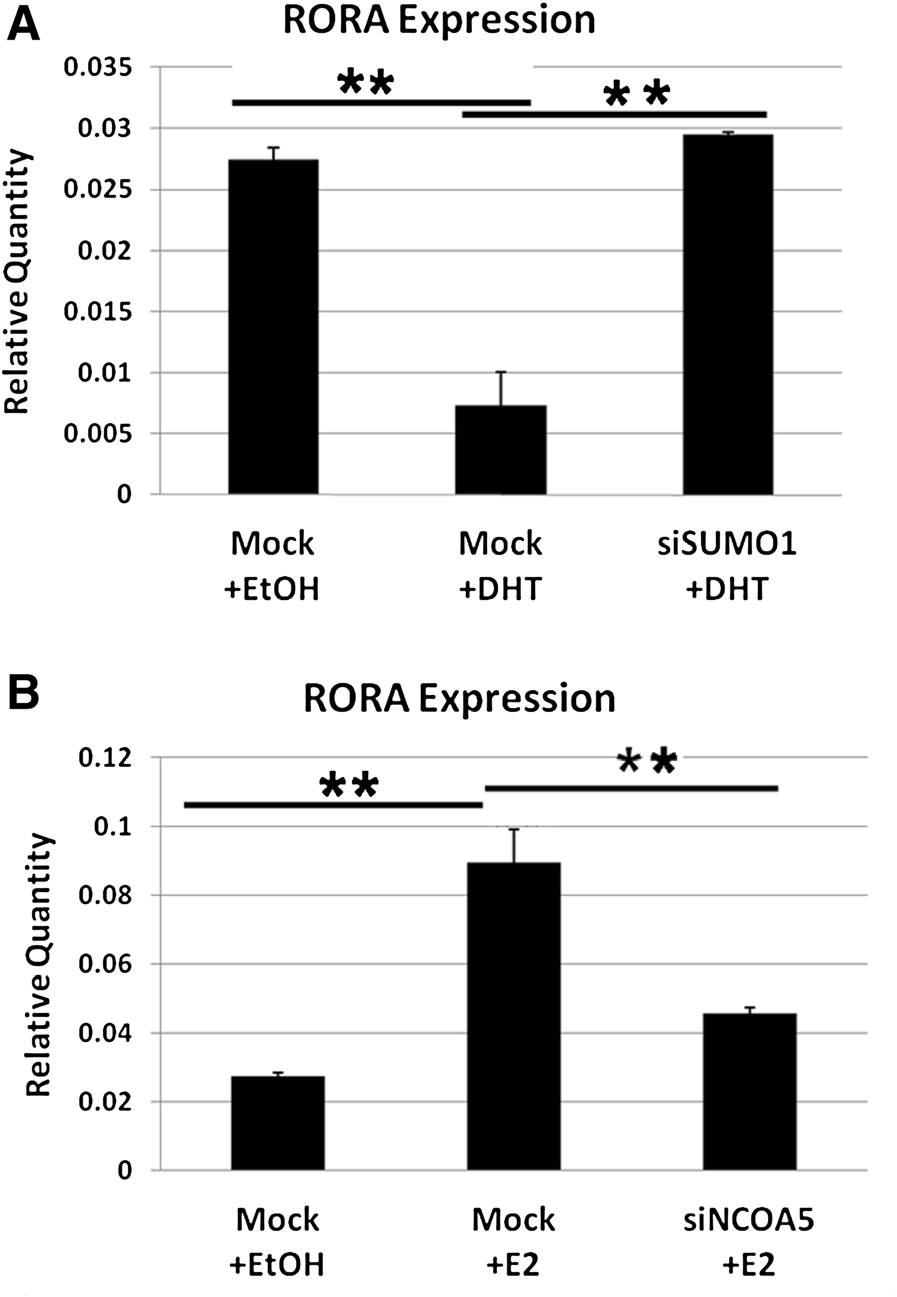
**siRNA-mediated knockdown of SUMO1 and NCOA5 show functional involvement of these coregulators in the regulation of *RORA *expression by AR and ERα, respectively. (A)** SH-SY5Y cells were transfected with siSUMO1 or vehicle control for 24 hours and treated with 1 nM DHT or ethanol for 2 hours. *RORA* expression was then measured by qRT-PCR analysis (n = 3). Relative *RORA* quantity in each sample was calculated using a standard curve obtained from *18S* expression levels in 10-fold serial dilutions of that sample. **(B)** NCOA5 knockdown was performed in the same manner as for SUMO1 and cells were treated with 1 nM E2. Error bars indicate SEM. Statistical significance of the differences between groups was determined by ANOVA (*P* <0.001 for each knockdown experiment) followed by post hoc *t* tests. **P <0.01. AR, androgen receptor; DHT, 4,5α-dihydrotestosterone; E2, 17β-estradiol; ERα, estrogen receptor alpha; SEM, standard error of the mean.

To further confirm that SUMO1 is required for DHT-mediated regulation of *RORA*, *SUMO1* expression in SH-SY5Y cells was suppressed using siSUMO1 and the transfected cells were then treated with 1 nM DHT. Using qRT-PCR analysis to measure *RORA* expression in siRNA-transfected cells, we found that the suppressive effect of DHT on *RORA* expression was completely abolished in cells transfected with siSUMO1 (Figure 5A), indicating that SUMO1 is required for DHT-mediated suppression of *RORA*. Similarly, we conducted siNCOA5 transfection and E2 treatment to confirm that NCOA5 is involved in E2-mediated regulation of *RORA*. The enhancing effect of E2 on *RORA* expression was significantly reduced (but not completely) in siNCOA5-transfected cells (Figure 5B), indicating that NCOA5 is involved in E2-mediated upregulation of *RORA*.

### Identification of RORA coregulators involved in regulation of *CYP19A1*

Inasmuch as RORA is also known to interact with coregulators to regulate the transcription of target genes, we therefore sought to determine whether the coregulators that we selected for this study are also involved in RORA-mediated regulation of gene transcription in human neuronal cells. We have recently demonstrated that RORA can potentially regulate the transcription of more than 2500 genes enriched for neurological functions implicated in ASD, and further validated several ASD-relevant genes, including *A2BP1*, *CYP19A1*, *HSD17B10*, *ITPR1*, *NLGN1*, and *NTRK2,* as transcriptional targets of RORA [38]. In this study, we investigated coregulator involvement in RORA-mediated regulation of *CYP19A1* because we have previously demonstrated that RORA protein is recruited to the promoter region of *CYP19A1*[16], *RORA* overexpression causes an increase in *CYP19A1* expression [16], and the expression of CYP19A1 (aromatase) protein is significantly reduced in brain tissues from ASD individuals as well as highly correlated with that of RORA [16].

**Figure 6:**
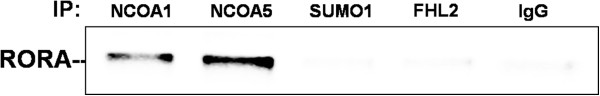
**Co-immunoprecipitation analysis of RORA coregulators.** Whole-cell lysates of SH-SY5Y cells were prepared and immunoprecipitated with anti-NCOA1, anti-NCOA5, anti-SUMO1, anti-FHL2, or IgG antibody. Western blot analysis was used to determine the enrichment of RORA protein in the immunoprecipitates.

**Figure 7:**
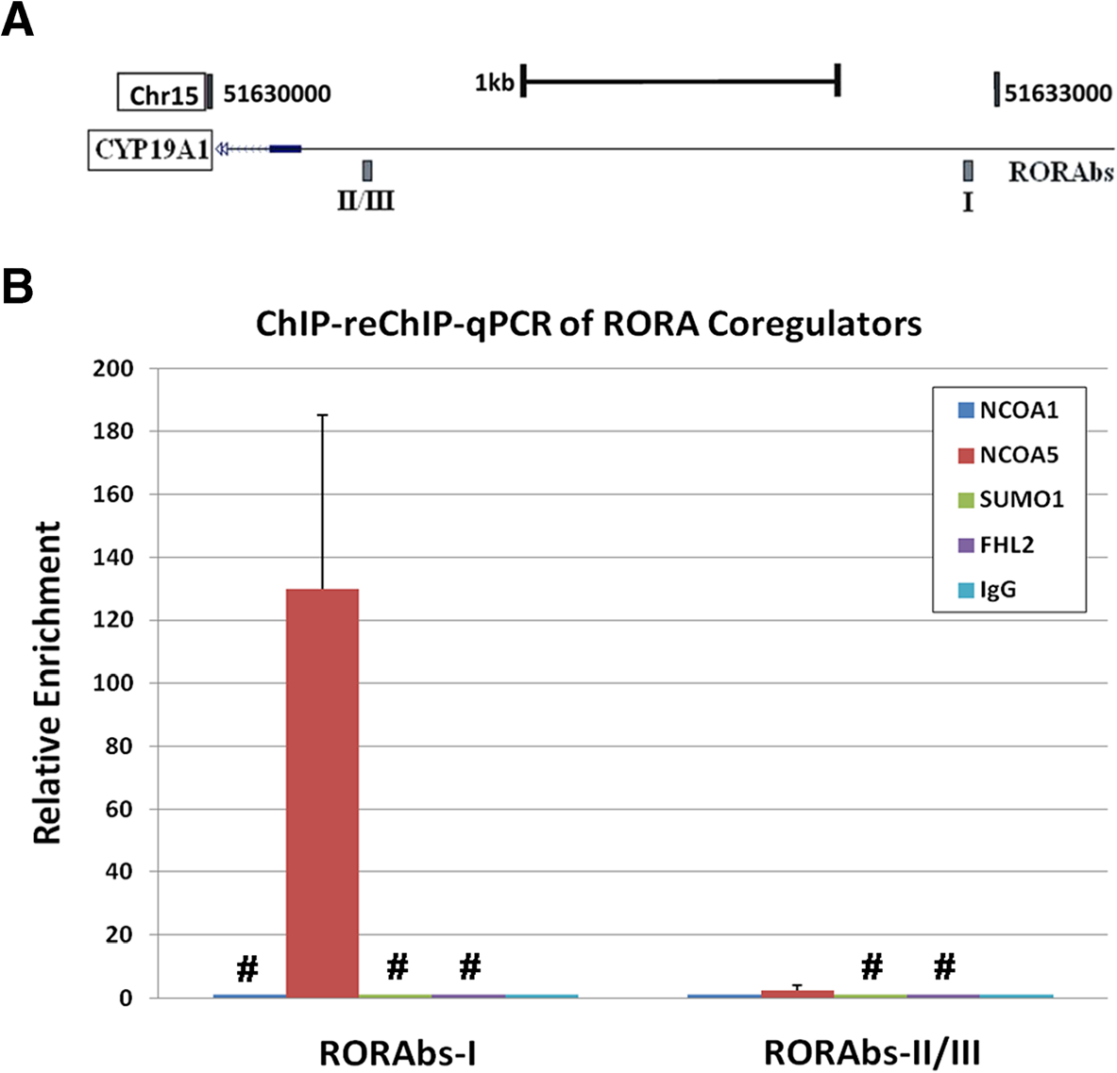
**ChIP-reChIP analysis of RORA coregulators binding to the *CYP19A1 *promoter region. (A)** Schematic diagram showing the upstream region of the CYP19A1 gene (edited from the UCSC Genome Browser). Potential RORA binding sites are labeled (RORAbs = RORA potential binding site). **(B)** Sequential chromatin immunoprecipitation (ChIP-reChIP) followed by qPCR analysis was conducted to determine whether NCOA1, NCOA5, SUMO1, or FHL2 interact with RORA in the *CYP19A1* promoter region. RORAbs-II and III are located adjacent to each other and cannot be analyzed separately by qPCR analysis, and are thus shown as RORAbs-II/III. Chromatin was isolated from SH-SY5Y cells and first immunoprecipitated with anti-RORA. The RORA-immunoprecipitated chromatin was washed to remove anti-RORA, then free chromatin was re-immunoprecipitated with anti-NCOA1, anti-NCOA5, anti-SUMO1, anti-FHL2, or nonspecific IgG, and a no-second-antibody control was also included. The enrichment of RORA binding sites in the *CYP19A1* promoter region in each re-ChIP sample was then determined using qPCR analysis (n = 3) and normalized with reference to the no-second-antibody control. Error bars indicate SEM. #Undetectable. ChIP-reChIP, sequential chromatin immunoprecipitation; SEM, standard error of the mean.

We first determined whether RORA interacts with the coregulators NCOA1, NCOA5, SUMO1, and FHL2 in SH-SY5Y cells by co-immunoprecipitation followed by western blot analysis of RORA protein. Figure 6 shows that RORA is enriched in protein samples immunoprecipitated with NCOA1 and NCOA5, indicating that RORA interacts with these coregulators in the human neuronal cell line SH-SY5Y. To determine whether these coregulators are also involved in regulation of the *CYP19A1* gene, we conducted sequential chromatin immunoprecipitation using anti-RORA or IgG antibody, followed by reChIP using anti-NCOA1, anti-NCOA5, anti-SUMO1, anti-FHL2, or IgG antibody. The enrichment of each potential binding site for RORA (RORAbs) on the *CYP19A1* promoter (Figure 7A) in the re-immunoprecipitated chromatin was determined by qPCR analysis. Figure 7B shows an increase in the average enrichment of RORAbs-I in the promoter region of *CYP19A1* when chromatin was sequentially immunoprecipitated by anti-RORA, followed by anti-NCOA5 antibody, indicating that RORA interacts with NCOA5 at this RORA binding site in the promoter region of *CYP19A1*.

## Discussion

We have previously shown that male and female hormones inversely regulate the expression of *RORA*, a nuclear receptor deficient in the brain and lymphoblastoid cell lines derived from a subset of individuals with ASD [12,15], with DHT reducing *RORA* expression and E2 increasing it [16]. Because the reduction of RORA protein in brain tissues from individuals with ASD was highly correlated with reduction of aromatase (CYP19A1) protein which enzymatically converts testosterone to estradiol, these results suggested a molecular explanation for the observed increase in testosterone that has been associated with autistic traits [6-8,48-50]. On the other hand, we hypothesized that females might be more protected against RORA deficiency due to higher levels of E2 that stimulate *RORA* expression, thus reducing risk for ASD relative to males. However, the mechanisms for the suppression of *RORA* expression by DHT and the upregulation by E2 were unknown. This study was undertaken to investigate the mechanisms through which the sex hormones regulate RORA and, in particular, to identify the molecular determinants for the opposite regulation by male and female hormones. Moreover, having recently validated *CYP19A1* as a transcriptional target of RORA [38], we further investigated the mechanism of *CYP19A1* regulation by RORA.

### Involvement of AR and ER in the transcriptional regulation of RORA

Because androgens and estrogens can mediate transcriptional changes without directly involving their respective hormone receptors, we first sought to determine whether or not AR and ER were directly involved in the regulation of *RORA*. The results presented here demonstrate the direct involvement of both hormone receptors in the transcriptional regulation of *RORA*, and furthermore identify specific hormone receptor binding sites within the extended 10 kb region upstream of the *RORA* TSS that participate in the up- or downregulation of *RORA* expression by the hormones acting upon a *RORA*-promoter-driven luciferase reporter construct. Interestingly, male and female hormones can exert both stimulatory and inhibitory effects on luciferase expression, depending on the presence of specific hormone receptor binding sites within the *RORA* promoter construct. Because hormone receptors are known to regulate their target genes in association with either coactivator or corepressor proteins [51,52], we then investigated coregulator involvement in AR- and ER-mediated regulation of *RORA* within the SH-SY5Y neuronal cell model, focusing on four coregulators that were found to be differentially expressed in the severely language-impaired subtype of ASD that was also deficient in RORA [15].

### Identification of coregulator partners of AR and ER in the regulation of RORA

Here we show by co-immunoprecipitation that several coregulators among the four examined can associate with AR and ERα in neuron-like SH-SY5Y cells. These included NCOA1, NCOA5, and SUMO1 associations with AR, and NCOA5 and FHL2 (to a lesser extent) associations with ERα. We then used ChIP-reChIP assays to interrogate associations of these four coregulator proteins with AR and ERα on specific hormone receptor binding sites on the *RORA* promoter. With respect to AR associations, SUMO1 showed the greatest enrichment relative to the no-second-ChIP-antibody control, and this enrichment was specific for ARbs-I. However, the enrichment was not statistically significant (*P* >0.05), most likely due to the low expression level of AR in these cells which were originally derived from a female. To increase the sensitivity of detecting enrichment of the ARbs-I in the ChIP-reChIP experiment involving SUMO1, DNA resulting from the sequential chromatin immunoprecipitation was amplified using primers designed specifically against ARbs-I, and gel electrophoresis of the PCR product was performed to confirm enrichment of ARbs-I in the sequential pull down using antibodies against AR and SUMO1 in comparison to that obtained with control IgG. Although SUMO1 is frequently found in covalent attachment to its targets, it is also considered a coregulator, according to the Nuclear Receptor Signaling Atlas (http://www.nursa.org) [53,54], that is often associated with transcriptional repression [55,56]. Indeed, SUMO1 associations with AR have been reported to modulate the transcriptional activity of AR [57]. In contrast to coregulator interactions with AR on the *RORA* promoter, ERα was found to significantly associate with NCOA5, a reported coactivator of ERα [58], at ERbs-I, while FHL2 was found to significantly associate with ERα at ERbs-IV. Interestingly, unlike other coregulators, NCOA5 does not require the ligand-dependent activation function-2 (AF2) domain of the target nuclear receptors for interaction, and can form complexes with both ERα and ERβ in the absence of ligand. On the other hand, FHL2 can exhibit dual coregulatory functions, acting as a corepressor of ERα and ERβ [59] and a coactivator of AR [60]. The differential associations of these two coregulators at different ER binding sites on the *RORA* promoter are interesting in light of the luciferase assays that revealed that the enhancing effect of E2 was manifested only when ERbs-I was present (on the 10 kb promoter construct). In the absence of ERbs-I and NCOA5 binding to the *RORA* promoter, E2 had a repressive effect on *RORA* expression, possibly due to the binding of the ER corepressor FHL2 on the most proximal ER binding site, ERbs-IV.

To determine the functional role of SUMO1 in the repression of *RORA* by DHT, we used siRNA against SUMO1 to reduce its expression in SH-SY5Y cells, and then monitored *RORA* expression in the presence and absence of DHT. The suppression of *RORA* expression by DHT treatment was completely abolished in the presence of siSUMO1, but not in the mock-treated control. Similarly, we monitored E2-mediated enhancement of *RORA* expression in siNCOA5-treated and mock-treated cells and found that siNCOA5 significantly reduced the upregulation of *RORA* expression by E2, but not completely, most likely due to incomplete knockdown of NCOA5 by siNCOA5 (as shown in Additional file 5). Alternatively, other untested coregulators may be involved in the E2-induced increase in RORA expression. Collectively, these results suggest that the regulation of *RORA* by androgen and estrogen is complex and dependent not only on the binding of the respective hormone receptors to specific hormone receptor binding sites on the *RORA* promoter, but also on the recruitment of specific coregulators to the hormone receptors.

### Identification of a coregulator in RORA-mediated regulation of CYP19A1

Inasmuch as we have demonstrated that RORA is a nuclear hormone receptor that transcriptionally regulates *CYP19A1*[38], we were interested in identifying coregulators that associate with RORA on the *CYP19A1* promoter. Co-immunoprecipitation analyses using antibody against each of the four coregulators followed by western blot analyses for presence of RORA in the immunoprecipitates demonstrated that both NCOA1 and NCOA5 are capable of associating with RORA in SH-SY5Y cells, while SUMO1 and FHL2 do not. However, ChIP-reChIP analyses performed with anti-RORA antibody followed, in separate immunoprecipitations, by antibodies against each of the four coregulators showed that only NCOA5 could interact with RORA on the *CYP19A1* promoter, and that the promoter binding involved only the more distal RORAbs-I. These studies suggest that NCOA5 can serve as a coregulator of both RORA and ERα within neuronal cells. This coincidence is interesting inasmuch as RORA and ER share the same consensus binding sites on DNA, AGGTCA, suggesting the overlap of at least some of their transcriptional targets. As suggested earlier, the existence of shared gene targets (including *RORA*) may in part compensate for RORA deficiency in females who, with higher levels of estrogen, exhibit lower susceptibility to ASD.

### Relevance of these findings to the sex bias in ASD

**Figure 8:**
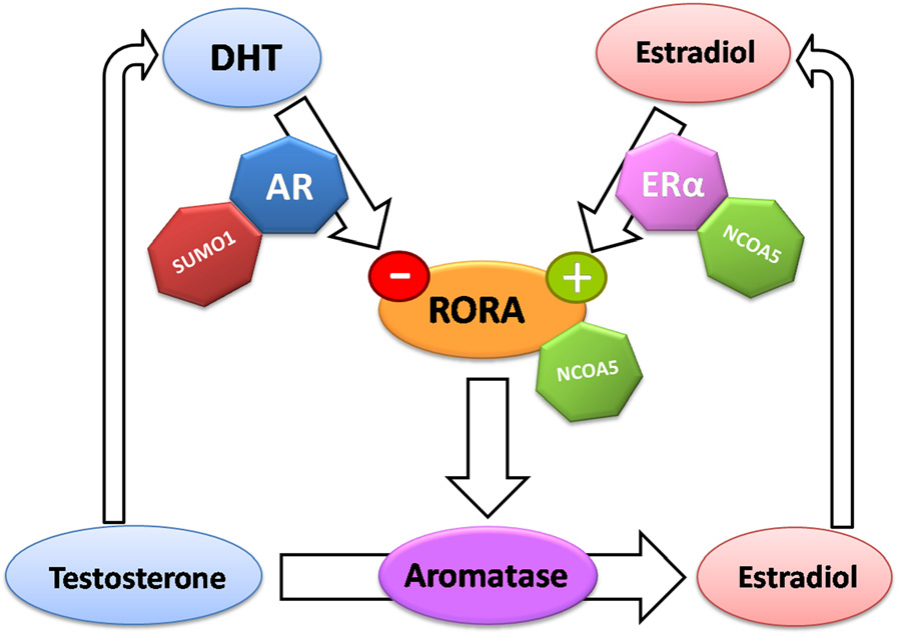
**A model for the opposite effects of male and female hormones on *RORA *expression.** The schematic illustrates a mechanism through which the observed reduction in RORA in the autistic brain may lead to increased testosterone levels through downregulation of aromatase. Our model suggests that AR, in association with SUMO1 functioning as a corepressor, is directly involved in the suppression of *RORA* expression by testosterone, whereas ERα association with the coactivator NCOA5 is involved in the upregulation of *RORA* expression by estradiol. RORA, in turn, positively regulates expression of aromatase, probably by interacting with NCOA5. AR, androgen receptor; ERα, estrogen receptor alpha.

Figure 8 presents a working model that integrates the results of these studies with those of our earlier studies that demonstrated the opposite regulation of *RORA* by male and female hormones and the regulation of *CYP19A1* by RORA [16,38]. In this model, a reduction in *RORA* expression, which may be induced by increased methylation, which we have demonstrated previously in cell lines from individuals with ASD [12] is expected to lead to a decrease in *CYP19A1* (aromatase), which, in turn, would result in the accumulation of its substrate testosterone. The highly active metabolite of testosterone, DHT, can then further suppress *RORA* expression, exacerbating RORA deficiency. Here, we show that the corepressor SUMO1 collaborates with AR in mediating the downregulation of *RORA*. Thus, an increase in SUMO1 expression, which we have detected in LCL from individuals with ASD relative to typical controls [15], may reinforce the androgen-mediated downregulation of *RORA*. Conversely, the model predicts that estradiol, the product of the aromatase reaction, is expected to decrease with the reduced expression of *CYP19A1*, and lower E2 would therefore dampen *RORA* expression. This study shows that NCOA5 is a strong coactivator of ERα at the ERbs-I on the *RORA* promoter. Interestingly, the expression of *NCOA5* is the most reduced among the differentially expressed coregulators in LCL derived from individuals with ASD [15]. Our study suggests that a reduction in this coactivator would further dampen *RORA* transcription via ER. The net outcome of the molecular changes and associations that we have identified in several studies regarding the hormonal regulation of *RORA* (and its transcriptional target, *CYP19A1*), their respective nuclear receptors and associated coregulators suggests that the aberrant expression of any of the genes in this interacting network in the directions that have been observed in our studies on cells and tissues from individuals with ASD can lead to increased testosterone, which has been linked to increased autistic traits [7,8,50]. Furthermore, our studies provide a plausible explanation for lower female susceptibility to ASD due to the positive effects of estrogen on *RORA* expression, which offers a buffer against conditions leading to RORA deficiency.

### Implications of coregulator involvement in the polygenicity of ASD

This is the first study to demonstrate the involvement of coregulators in the hormonal regulation of a functionally relevant autism candidate gene, *RORA*, whose deficiency can impact multiple processes that are known to be disrupted or impaired in ASD, such as synaptogenesis, axon guidance, dendritic extension, neurotransmission, circadian regulation, and higher level functions, such as learning and speech [38]. Because coregulators interact in combinatorial fashion with different nuclear hormone receptors to modulate gene expression, often in response to endogenous as well as exogenous agents, coregulators have been proposed to be ‘key integrators of environmental signals’ and are thus likely contributors to the polygenic nature of complex diseases [61]. This study shows that the response of *RORA* to androgens and estrogens depends on both the availability of specific AR and ER binding sites on the *RORA* promoter as well as the recruitment of specific coregulators to the respective hormone receptors on the promoter. Coregulator involvement in gene dysregulation in ASD thus represents a new paradigm in the investigation of ASD, which are clearly complex polygenic disorders with many genes already implicated by large-scale genetics, gene expression, and epigenetic studies.

### Study limitations and future directions

To gain additional insight into the molecular mechanisms involved in the regulation of *RORA* by sex hormones, we investigated the biochemical associations and functional involvement of hormone receptors and coregulator proteins in DHT- and E2-mediated down- and upregulation of *RORA*, respectively. However, we limited our study to selected coregulators that were differentially expressed in our previous expression profiling study of LCL from individuals with ASD rather than examine all possible coregulator interactions, as has been recently accomplished by proteomic analysis of coregulators of ERα which was targeted against a synthetic DNA template containing four tandem estrogen response elements fused to the adenovirus E4 gene promoter [62]. This comprehensive analysis of coregulator associations with ERα in nuclear extracts of MCF7 and HeLa cells revealed as many as 17 coregulators that associated with the hormone receptor on the DNA template. Interestingly, this study also revealed that the associations could rapidly change in response to phosphorylation of the ERα-coregulator complexes, revealing the dynamic nature of coregulator binding to such complexes. Thus, our study provides only a restricted analysis of the possible coregulator associations with ERα and AR that can modulate *RORA* expression in response to sex hormones, and we cannot rule out the involvement of other coregulators not studied here. Furthermore, inasmuch as coregulator recruitment is known to be tissue specific [63], it will be of interest to investigate coregulator-hormone receptor complexes in the brain of individuals with ASD vs. that of typical individuals.

Another limitation is that this study focuses only on ERα, while both ERα and ERβ are known to be ubiquitously expressed in the human brain throughout life. However, there is evidence that ERα may be more important in biological functions associated with autism, including early cortical development processes [64], regulation of transcriptional targets in the cortex [64,65], neuroprotection against cytotoxicity [66] and ischemia [67], and social discrimination [68]. Nevertheless, the mechanisms through which ERβ may be involved in regulation of *RORA* deserve further study since ERβ is also known to be highly expressed in cortex, amygdala, and cerebellum, where AR is also highly expressed [69]. Moreover, ERβ may be more important for development of future therapies addressing RORA deficiency because ERβ is known to have little or no expression in the breast or uterus. Thus, selective activation of ERβ may provide the beneficial effects of ER signaling in the brain without undesired effects in reproductive organs.

## Conclusions

In summary, we show that AR and ERα are respectively involved in the suppression and enhancement of *RORA* expression by male and female hormones in a neuronal cell model, and that the corepressor SUMO1 is needed for AR-mediated suppression, while the coactivator NCOA5 is involved in the ER-mediated upregulation of *RORA*. We further demonstrate that NCOA5 can interact with RORA on the promoter of *CYP19A1*, revealing another similarity in gene regulatory mechanisms between RORA and ER which share the same DNA consensus binding sites. Finally, we show for the first time, the involvement of coregulators, when aberrantly expressed, as potential contributors to the polygenic nature of gene dysregulation in ASD.

## Abbreviations

A2BP1: Ataxin 2 binding protein 1 (also known as RBFOX1); AR: Androgen receptor; ASD: Autism spectrum disorder; BCA: Bicinchoninic acid; ChIP: Chromatin immunoprecipitation; ChIP-reChIP: Sequential chromatin immunoprecipitation; Co-IP: Co-immunoprecipitation; CYP19A1: Cytochrome P450 family 19 subfamily A polypeptide 1; DHT: 4,5α-dihydrotestosterone; (D)MEM: Dulbecco’s modified Eagle’s medium; E2: 17β-estradiol; EDTA: Ethylenediaminetetraacetic acid; ERα: Estrogen receptor alpha; ERβ: Estrogen receptor beta; FHL2: Four and a half LIM domains 2; HRP: Horseradish peroxidase; HSD17B10: Hydroxysteroid (17-beta) dehydrogenase 10; IPTG: Isopropyl-β-D-thio-galactoside; ITPR1: Inositol 1,4,5-trisphosphate receptor type 1; LB: Luria-Bertani medium; LCL: Lymphoblastoid cell line; NCOA1: Nuclear receptor coactivator 1; NCOA5: Nuclear receptor coactivator 5; NLGN1: Neuroligin 1; NTRK2: Neurotrophic tyrosine kinase receptor type 2; PBS: Phosphate-buffered saline; PMSF: Phenylmethylsulfonyl fluoride; PVDF: Polyvinylidene fluoride; qRT-PCR: Quantitative reverse transcriptase polymerase chain reaction; qPCR: Quantitative polymerase chain reaction; RORA: Retinoic acid receptor-related orphan receptor alpha; SEM: Standard error of the mean; SDS: Sodium dodecyl sulfate; SH-SY5Y: Human neuroblastoma cell line; siRNA: Silencing RNA; SUMO1 SMT3: Suppressor of mif two 3 homolog 1; TBST: Tris-buffered saline and Tween 20; TSAP: Thermosensitive alkaline phosphatase; TSS: Transcription start site; X-Gal: 5-bromo-4-chloro-indolyl-β-D-galactopyranoside.

## Competing interests

The authors declare that they have no competing interests.

## Authors’ contributions

TS and VWH conceived of the study, contributed to the study design, performed the data analyses, and prepared the manuscript. TS conducted the experiments. Both authors read and approved the final manuscript.

## Supplementary Material

Additional file 1 List of antibodies and siRNAs used in this study. Click here for file

Additional file 2 Transfection efficiency of siRNAs. Click here for file

Additional file 3 List of primers for PCR cloning, ChIP-reChIP-qPCR, and qRT-PCR analyses. Click here for file

Additional file 4 **Transcription factor binding sites in promoter regions of *RORA *** **and *CYP19A1 *** **genes.** Click here for file

Additional file 5 Nuclear receptor coregulators found to be dysregulated in LCLs from individuals with ASD. Click here for file
