# Supplementary material for: Differential recruitment of coregulators to the RORA promoter adds another layer of complexity to gene (dys) regulation by sex hormones in autism
Source: Mol Autism. 2013 Oct 11;4:39. doi: 10.1186/2040-2392-4-39 (PMC4016566; doi:10.1186/2040-2392-4-39)
Supplement: Additional file 1 — List of antibodies and siRNAs used in this study. [file 2040-2392-4-39-S1.doc]

**Additional file 1. List of antibodies and siRNAs used in this study.**

| **Description** | **Species** | **Type** | **Catalog No** |
| --- | --- | --- | --- |
| **Antibodies** |  |  |  |
| Anti-AR (441) | mouse | monoclonal | SC-7305 |
| Anti-ERα (D-12) | mouse | monoclonal | SC-8005 |
| Anti-FHL2 (C-16) | goat | polyclonal | SC-13409 |
| Anti-NCOA1 (C-20) | goat | polyclonal | SC-6096 |
| Anti-NCOA5 (G-20) | goat | polyclonal | SC-86178 |
| Anti-RORA (H-65) | rabbit | polyclonal | SC-28612 |
| Anti-SUMO1 (N-19) | goat | polyclonal | SC-6376 |
| Normal goat IgG | goat |  | SC-2028 |
| Normal rabbit IgG | rabbit |  | SC-2027 |
| Normal mouse IgG | mouse |  | SC-2025 |
| Anti-mouse IgG-HRP | donkey |  | SC-2318 |
| Anti-rabbit IgG-HRP | donkey |  | SC-2317 |
| Anti-goat IgG-HRP | donkey |  | SC-2020 |
|  |  |  |  |
| **siRNAs** |  |  |  |
| AR siRNA (h) |  |  | SC-29204 |
| ERα siRNA (h) |  |  | SC-29305 |
| SUMO1 siRNA (h) |  |  | SC-29498 |
| NCOA5 siRNA (h) |  |  | SC-75885 |

All antibodies and siRNAs were purchased from Santa Cruz Biotechnology, Inc. (Dallas, TX, USA).
