# Supplementary figures and images for: Differential recruitment of coregulators to the RORA promoter adds another layer of complexity to gene (dys) regulation by sex hormones in autism
Source: Mol Autism. 2013 Oct 11;4:39. doi: 10.1186/2040-2392-4-39 (PMC4016566; doi:10.1186/2040-2392-4-39)

**Additional file 2. Transfection efficiency of siRNAs**

**
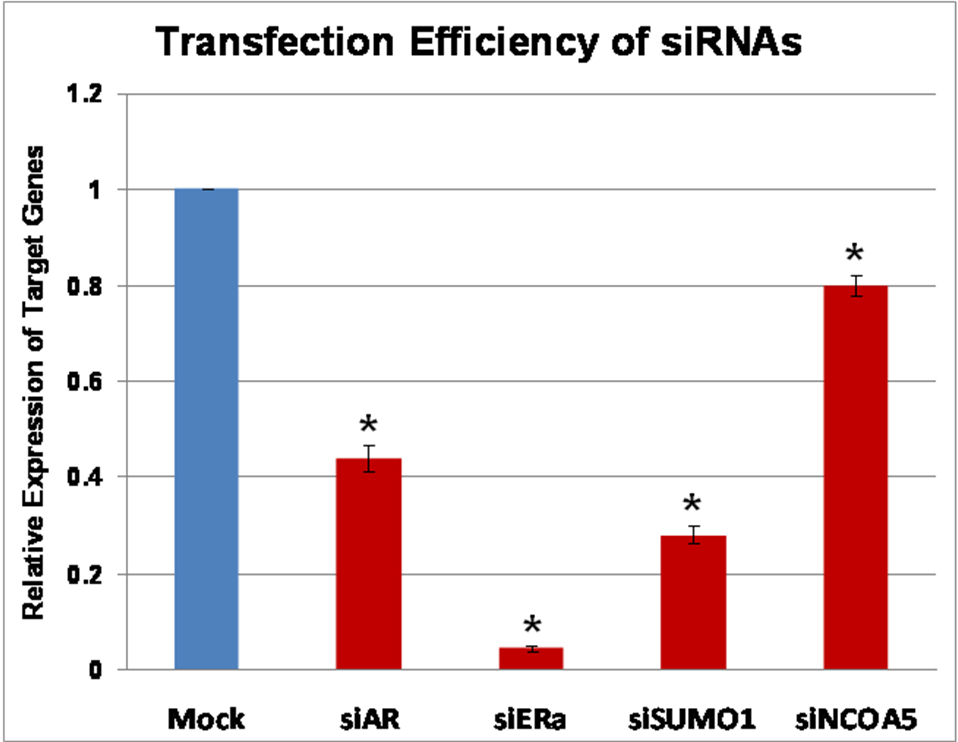
**

Supplement: Additional file 2 — Transfection efficiency of siRNAs. [file 2040-2392-4-39-S2.doc]
