# Supplementary material for: Differential recruitment of coregulators to the RORA promoter adds another layer of complexity to gene (dys) regulation by sex hormones in autism
Source: Mol Autism. 2013 Oct 11;4:39. doi: 10.1186/2040-2392-4-39 (PMC4016566; doi:10.1186/2040-2392-4-39)
Supplement: Additional file 3 — List of primers for PCR cloning, ChIP-reChIP-qPCR, and qRT-PCR analyses. [file 2040-2392-4-39-S3.doc]

**Additional file 3. List of primers for PCR cloning, ChIP-reChIP-qPCR, and RT-qPCR analyses.**
