# Supplementary material for: Differential recruitment of coregulators to the RORA promoter adds another layer of complexity to gene (dys) regulation by sex hormones in autism
Source: Mol Autism. 2013 Oct 11;4:39. doi: 10.1186/2040-2392-4-39 (PMC4016566; doi:10.1186/2040-2392-4-39)
Supplement: Additional file 4 — Transcription factor binding sites in promoter regions of RORA and CYP19A1 genes. [file 2040-2392-4-39-S4.doc]

**Additional file 4. Transcription factor binding sites in promoter regions of *RORA* and *CYP19A1* genes.**
