# Supplementary material for: Differential recruitment of coregulators to the RORA promoter adds another layer of complexity to gene (dys) regulation by sex hormones in autism
Source: Mol Autism. 2013 Oct 11;4:39. doi: 10.1186/2040-2392-4-39 (PMC4016566; doi:10.1186/2040-2392-4-39)
Supplement: Additional file 5 — Nuclear receptor coregulators found to be dysregulated in LCLs from individuals with ASD. [file 2040-2392-4-39-S5.doc]

**Additional File 5. Nuclear receptor coregulators found to be dysregulated in LCLs from individuals with ASD**

**
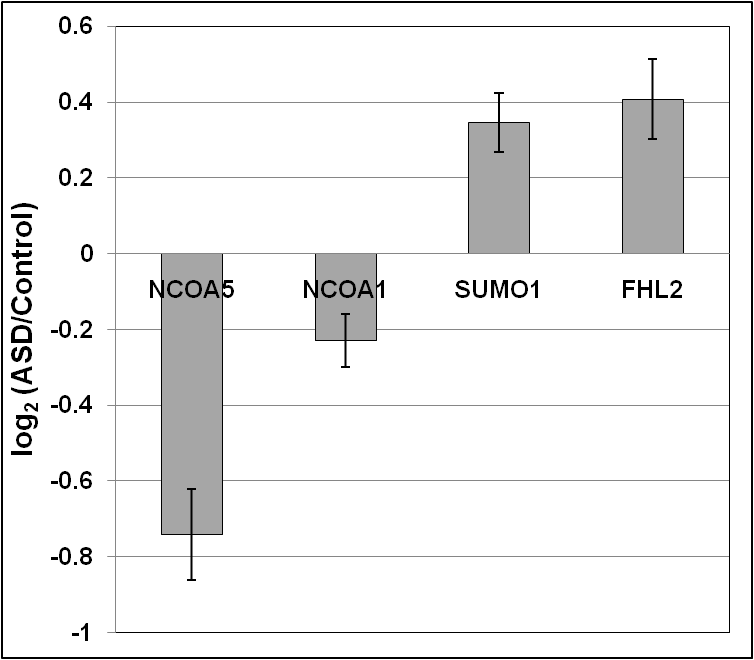
**
